# Porcine Reproductive and Respiratory Syndrome virus (PRRSv): A Cross-Sectional Study on ELISA Seronegative, Multivaccinated Sows

**DOI:** 10.3390/v14091944

**Published:** 2022-08-31

**Authors:** Jorian Fiers, Marylène Tignon, Ann Brigitte Cay, Xavier Simons, Dominiek Maes

**Affiliations:** 1 Unit Viral Re-emerging, Enzootic and Bee diseases, Department Infectious diseases in animals, Sciensano, Groeselenbergstraat 99, 1066 Ukkel, Belgium; 2 Unit of Porcine Health Management, Department of Reproduction, Obstetrics and Herd Health, Faculty of Veterinary Medicine, Ghent University, Salisburylaan 133, 9820 Merelbeke, Belgium; 3 Unit Veterinary Epidemiology, Department Epidemiology and Public Health, Sciensano, Rue Ernest Blerot 1, 1070 Anderlecht, Belgium

**Keywords:** PRRSv, vaccination, immunology, sow, non-responder

## Abstract

Vaccination against Porcine Reproductive and Respiratory Syndrome virus (PRRSv) is widely used to control clinical disease, but the effectiveness appears in some cases to be suboptimal. Field reports have stated the presence of routinely PRRSv-vaccinated but ELISA seronegative sows: the ELISA non-responders. The real extent of this phenomenon (prevalence–origin–consequences) was not yet investigated. In this study, the prevalence of ELISA non-responders was assessed by measuring PRRSv-specific antibodies in 1400 sows, originating from 70 PRRSv-vaccinating sow herds, using IDEXX ELISA (ELISA 1) and CIVTEST E/S ELISA (ELISA 2). Neutralizing antibodies (NAbs) were quantified in a virus neutralization assay. Univariable logistic regression was used to identify herd risk factors for the presence of ELISA non-responders. The global prevalence of non-responders varied from 3.5% (ELISA 1) to 4.1% (ELISA 2), the herd-level prevalence was 40% and the within-herd prevalence ranged from 5% to 20% (ELISA 1) and from 5% to 30% (ELISA 2). The ELISA non-responders had significantly lower NAbs than the ELISA responders. Herds using the combination of one modified live vaccine and one killed vaccine had a significantly reduced risk of having ELISA non-responders. A first assessment of the prevalence and possible consequences of ELISA non-responders has been provided by this study. The clinical importance, origin and underlying immunological mechanisms warrant further research.

## 1. Introduction

Porcine Reproductive and Respiratory Syndrome virus (PRRSv), an enveloped, positive-sense, single-stranded RNA virus with a genome of approximately 15 kb in length, is the causative agent of one of the most devastating diseases in the worldwide swine industry. The virus belongs to the order *Nidovirales,* family *Arteriviridae*, subfamily *Variarterivirinae*, genus *Betaarterivirus*. Two different species of PRRSv have been described: *Betaarterivrus suid 1* (PRRSv-1) and *Betaarterivrus suid 2* (PRRSv-2) [1]. Nucleotide differences between both species can be as large as 60%. Furthermore, within each species, a whole range of subtypes have been described, emphasizing the genetic diversity of this small virus [2,3]. PRRSv has a narrow in vivo cell tropism, infecting cells of the monocyte and macrophage lineage, with a preference to infect subsets of differentiated macrophages in lung, placenta and lymphoid tissue [4]. Disease manifestations of PRRSv infection reflect this tropism, with clinical signs varying from reproductive failure to respiratory disease. In PRRS-affected gilts and sows, an increased incidence of aborted fetuses, irregular returns to estrus and the birth of weak, premature and dead piglets have been reported. Infected weaned piglets and fattening pigs show symptoms of respiratory distress, including coughing, sneezing and dyspnea [5].

Since PRRSv infection can lead to reproductive failure in gilts and sows and to performance losses in piglets and grow-finishing pigs, it is the major cause of economic losses in the worldwide swine industry. Economic models estimate the annual cost of productivity losses due to PRRS in all national breeding and growing-pig herds in the United States to be USD 664 million [6]. In Europe, economic models estimate a median annual loss of EUR 127 to EUR 650 per sow in a farrow-to-finish herd of 1000 sows when these sows are slightly affected by PRRS in only the reproductive system or severely affected in both the respiratory and reproductive systems, respectively [7].

To date, PRRS control is hard to manage, and sow and/or piglet vaccination against PRRSv is widely used for prevention and control of the disease. Unfortunately, the effectiveness of PRRSv vaccination is often unpredictable and suboptimal, with PRRS outbreaks occurring despite routine vaccination being practiced [8]. Despite the suboptimal effectiveness, PRRSv vaccination of the sow and/or piglet population has clear welfare and economic benefits, with European models estimating the annual benefit of PRRSv sow vaccination to be EUR 150/sow, even with a low vaccination effectiveness of 50% and a high price of EUR 1.50 per vaccine dose [9]. The effectiveness of PRRSv vaccination in field conditions depends on multiple factors including the vaccine, the vaccination scheme, the infection level in the farm, the type of field strain(s) present on the herd and the biosecurity measures in the farm [8,10].

Two large classes of PRRSv vaccines are used: modified live vaccines (MLVs) and inactivated/killed vaccines (KVs). MLVs offer complete and partial protection against homologous and heterologous strains, respectively, but have some safety concerns including the possible reversion to virulence and the risk of recombination between one MLV and a wild PRRSv strain or the recombination between two different MLVs. KVs offer only limited protection (especially in naïve animals) but are beneficial from a safety point of view [11,12,13,14,15,16,17,18].

In Belgium, several swine veterinarians have reported that pigs (both sows and piglets) often remain ELISA seronegative (ELISA non-responders) despite being vaccinated against PRRSv (data not published). This is also confirmed in several field and experimental studies, in which not all PRRSv-vaccinated animals seem to have detectable ELISA antibodies (Abs) [19,20,21,22]. In piglets, the presence of maternally derived Abs has been described to be a possible impairing factor for the immune responses after PRRSv vaccination [20,21], which could explain why some PRRSv vaccinated piglets remain ELISA seronegative. However, in routinely PRRSv-vaccinated sows this impairing factor is not present, and it should be expected that routinely vaccinated sows all have detectable PRRSv Abs. The real extent of routinely PRRSv-vaccinated but ELISA seronegative sows is unknown, and further investigation is needed to determine the prevalence, the origin and possible consequences of these ELISA non-responding sows.

The main objectives of this cross-sectional field study were to assess the global prevalence (proportion of ELISA non-responding sows), the herd-level prevalence (proportion of herds with ELISA non-responding sows) and the within-herd prevalence (proportion of ELISA non-responding sows per herd) in PRRSv-vaccinating sow herds in Belgium. Secondly, the presence of PRRSv-specific neutralizing antibodies (NAbs) was investigated in a selection of ELISA seronegative and seropositive sows, to obtain a first indication of the possible clinical importance of the ELISA non-responding sows. Finally, possible factors that could be associated with the presence of ELISA non-responders were assessed.

## 2. Materials and Methods

### 2.1. Study Design

Seventy sow herds, with at least 150 breeding sows that practiced routine PRRSv vaccination, were included in the study. Firstly, the proportion of sow herds (sow capacity > 150) within each province in Belgium was assessed using the National Animal Identification and Registration database (Sanitel database). Secondly, a similar proportion of sow herds in each province was selected, ensuring a geographically representative number of sow herds (Figure S1). According to the Sanitel database, there were 1099 active sow herds (sow capacity > 150) in Belgium at the moment of herd selection (October 2020). The sample population of this cross-sectional study included 70 out of these 1099 sow herds (6.4%). The median herd size of the total population was 260 sows (min: 150, max: 3900, IQR: 200), while the median herd size of the study population was 305 sows (min: 150, max: 2000, IQR: 231.5). At the moment of sampling, the number of sows present in the study’s herds did not differ much from the herd sizes reported in the Sanitel database, with a median number of 300 sows on site (min: 115, max: 2200, IQR: 200).

Selected herds were visited once between 21 October 2020 and 7 May 2021 by the principal investigator (PI) and the respective herd veterinarian. A total of 24 different herd veterinarians, belonging to 15 different veterinary practices, collaborated in this study. Within each herd, twenty breeding sows of different parities were selected for blood sampling. The parity distribution of the sampled sows was as follows: 274 (19.57%) first parity, 296 (21.14%) second parity, 267 (19.07%) third parity and 563 (40.21%) 4+ parity sows. The blood samples were taken by puncture of the vena jugularis externa using the Primavette V serum 7.5 mL blood collection system (KABE Labortechnik, Nümbrecht, Germany). Most sows were sampled in the farrowing unit. In a few farms, the herd owners preferred to sample the sows in the gestation unit. All blood samples were stored at 2 °C to 8 °C until transport. The samples were transported to the laboratory of Sciensano (Brussels) within a maximum of 3 days after sampling. Upon arrival in the laboratory, samples were immediately centrifuged (1000g—15 min—4 °C), and serum was collected and subsequently stored at −20 °C until analysis. All herds and samples were coded in an anonymous way using a random, unique identifier. Farm data were collected during the herd visit by means of two questionnaires. First, the BioCheck questionnaire (Biocheck.UGent^TM^, Gent, Belgium) was used to score the internal, external and total biosafety practices on each herd [23,24]. Second, a PRRSv-specific questionnaire, developed for this study, was used to gather data concerning general herd characteristics and information about PRRSv status, management and vaccination practices.

### 2.2. Enzyme-Linked Immunosorbent Assay (ELISA)

All 1400 samples (70 herds × 20 samples) were analyzed for the presence of PRRSv-specific Abs using commercially available ELISA kits. To avoid the influence of a certain ELISA kit effect, different commercial ELISAs were used to assess the possible non-responsiveness. Abs directed against recombinant PRRSv-1 and PRRSv-2 ORF7 antigens (nucleocapsid protein) were assessed using the IDEXX PRRS X3 Ab test (ELISA 1) (IDEXX Laboratories, Westbrook, Maine, United States), which is widely considered the gold standard for PRRSv Ab testing [5]. CIVTEST SUIS PRRS E/S test (ELISA 2) (HIPRA, Amer, Girona, Spain) was used for the detection of Abs directed against a specific PRRSv-1 antigen-glycoprotein-rich extract [25]. Seronegative samples, as well as seropositive samples just above the cut-off values, were retested once to confirm their seronegative/seropositive status. Ambiguous results were retested until a consensus result was achieved. Seronegative samples in either or both tests were further analyzed using the INgezim PRRS 2.0 test (ELISA 3) (Eurofins Technologies Ingenasa, Madrid, Spain) and the ID Screen PRRS Indirect ELISA kit (ELISA 4) (Innovative Diagnostics, Grabels, France) which both detect Abs against recombinant PRRSv-1 and PRRSv-2 ORF7 antigens, to confirm the seronegative status. All ELISA analyses were performed according to the manufacturers’ guidelines. The WellWash Versa Microplate Washer (Thermo Fisher Scientific, Waltham, MA, USA) was used for all washing steps, and the MultiSkan FC Microplate Photometer (Thermo Fisher Scientific, Waltham, MA, USA) was used to measure absorbance at 650 nm (ELISA 1) or 450 nm (ELISA 2, 3 and 4). Samples with a sample-to-positive (S/p) value ≥ 0.4 in ELISA 1, 3 and 4 were considered to be seropositive. Samples with a Relative Index Percent (IRPC value) > 20 in ELISA 2 were considered to be seropositive. An additional subdivision of seropositive samples was made for this study: samples having an S/p value between 0.4 and 0.6 were considered to be low-seropositive in ELISA 1, while samples with an IRPC value between 20 and 30 were considered to be low-seropositive in ELISA 2.

### 2.3. Virus Neutralization Assay (VN)

The presence of PRRSv-specific NAbs was assessed for a selection of 319 samples, including 81 ELISA seronegative samples (in either or both ELISA 1 and 2), 76 ELISA low seropositive samples (in either or both ELISA 1 and 2) and 162 randomly selected ELISA seropositive samples (in both ELISA 1 and 2), with at least two seropositive samples from each herd. VN was performed as previously described, with slight modifications [26]. In short, sera to test were first heat-inactivated by incubation at 56 °C for 30 min to ensure complement inactivation. In 96-well flat-bottom plates, sera were tested in duplicate by making two-fold serial dilutions (2^1^ to 2^8^) in Minimal Essential Medium (Thermo Fisher Scientific, Waltham, MA, USA) with antibiotics (MEM-A: MEM + 0.5% Penicillin, 0.1% Fungizone—250 µg/mL amphotericin B, 0.1% Gentamycin—50 mg/mL), followed by the addition of 50 TCID50 of the PRRSv-1 DV strain (propagated in MARC-145 cells) to each dilution. After incubation for 1 h at 37 °C (5% CO_2_), 100 µL of a MARC-145 cell suspension (10^5^ cells/mL in MEM-A + 10% FBS) was added to each well, and plates were incubated for at least 72 h at 37 °C (5% CO_2_). Following incubation, plates were washed 3 times in PBS and heat-fixed by two consecutive heating steps: 30 min at 37 °C and 1 h 20 at 80 °C. After fixation, cells were stained with the monoclonal PRRSv-specific mouse Ab 13 × 10^2^ (1/50 diluted in PBS + 10% goat serum—kindly provided by Prof. Dr. H. Nauwynck) as primary Ab, the HRP-conjugated polyclonal goat anti-mouse Ab (Agilent Technologies, Santa Clara, CA, USA) as secondary Ab and a chromogen substrate (19 mL Na-Acetate + 1 mL ACE + 10µL H_2_O_2_) for detection. For each sample, the VN titer in one run was determined as the Log_2_ of the highest dilution in which no staining was observed in one or both replicates. Each sample was tested in two separate runs, and the final VN titer was determined as the mean titer of both runs. Positive (only virus + cells) and negative (only cells + MEM-A) controls were included in each plate. Back titration of the viral stock was performed in each run to ensure homogeneity between different runs. Samples with a VN titer ≥ 2 Log_2_ were considered to be VN seropositive [27].

### 2.4. Statistical Analysis

GraphPad Prism 9 (GraphPad Software, San Diego, CA, USA) was used to visualize and analyze results. The hybrid Wilson/Brown method was used to calculate 95% confidence intervals (CI 95%) for proportions. The chi-square test was used to calculate differences in proportions of ELISA seronegative sows between different parities and to calculate differences in proportions of VN seropositive samples between ELISA seronegative and ELISA seropositive sows. Unpaired t-test was used to calculate differences in mean ELISA results between two different parities and to calculate differences in mean VN titers between ELISA low-seropositive and seropositive samples. One-way ANOVA and Tukey’s multiple comparisons test were used to calculate differences in mean ELISA results between all parities and to calculate differences in mean VN titers between ELISA seronegative, low-seropositive and seropositive samples. Unpaired t-test was used to compare differences in mean values of continuous variables (time since last PRRSv vaccination, Biosafety scores, herd size) between “seronegative” and “seropositive” herds. For this, herds having at least one ELISA 1 seronegative sow (on 20 sampled) were considered a “seronegative” herd, while herds without any ELISA 1 seronegative sow were considered a “seropositive” herd. Univariable logistic regression was used to investigate the association between categorical herd characteristics (potential risk factors), derived from the PRRSv-specific questionnaire, and the presence of at least one ELISA 1 seronegative sow (of 20 sampled) in the herd. Results of the univariable logistic regression are expressed as the odds ratio (OR) for having at least one ELISA 1 seronegative sow (of 20 sampled) for each variable compared to the reference variable. To reduce estimates’ bias, due to small number of events per variable, Firth’s correction was used when perfect separation was observed. The logistic regression was performed using R software (version 4.1.2). Results are shown as mean ± standard deviation (SD). *p*-values < 0.05 are considered to be significant.

## 3. Results

### 3.1. Results of the BioCheck Questionnaire

The internal biosecurity score for the study’s herds was 63.4 ± 13.5 (min: 35, max: 90), the external biosecurity score was 71.5 ± 8.5 (min: 53, max: 91), and the total biosecurity score was 67.7 ± 10.1 (min: 48, max: 87). Herds scored the lowest in subcategories “Feed, water and equipment supply”, “Location of the farm” and “Measures between compartments”. Highest scores were achieved in subcategories “Purchase of breeding pigs, piglets and semen”, “Finishing unit” and “Vermin and bird control” (Table 1).

### 3.2. Results of the PRRSv-Specific Questionnaire

Descriptive data concerning sow, gilt and piglet PRRSv vaccine management (Table 2) showed a high variety between herds, especially in the used gilt and sow PRRSv vaccine/PRRSv vaccine combinations and the used PRRSv sow vaccination scheme. Porcilis was the most frequently used sow vaccine in the selected herds (27/70, 38.6%), followed by the combination of Porcilis + Progressis (16/70, 22.9%), Unistrain (12/70, 17.1%) and Ingelvac MLV (6/70, 8.6%). Six other vaccines/vaccine combinations were used in the remaining nine herds. Six herds exclusively vaccinated their gilt and sow population with a PRRSv-2 MLV. Additionally, four herds vaccinated their gilt population with a PRRSv-2 MLV and their sow population with either a PRRSv-1 MLV, a PRRSv-1 MLV + a PRRSv-2 MLV or a PRRSv-2 MLV + a PRRSv-1 KV. Although PRRSv vaccination of the sow population is routinely practiced in all selected herds, 25/70 (35.71%) and 26/70 (37.14%) of the herd owners still reported the presence of clinical PRRS problems in the 12 months preceding the sample date, in their sow and piglet populations, respectively. Most common clinical signs in sows included abortions (18/25; 72%), premature birth (16/25; 64%) and stillborn/weak born piglets (10/25; 40%). In piglets, most common symptoms included coughing (18/26; 69.23%), sneezing (12/26; 46.15%) and removal from the nursery unit (11/26; 42.31%).

### 3.3. Presence of ELISA 1 Non-Responding Sows

**Figure 1:**
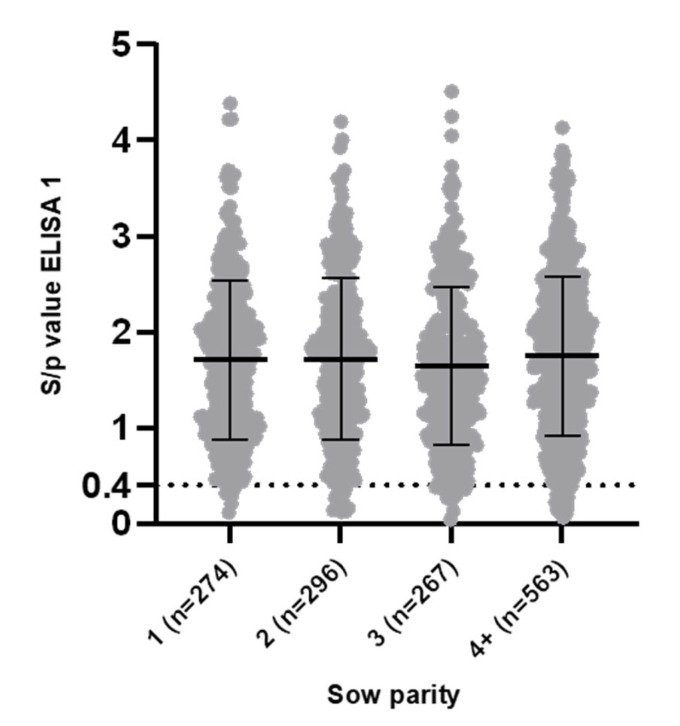
ELISA 1 (IDEXX PRRS X3) sample-to-positive (S/p) values in 1400 sow samples (parity 1 = 274, parity 2 = 296, parity 3 = 267 and parity 4+ = 563) originating from 70 Belgian sow herds that practice routine vaccination against Porcine Reproductive and Respiratory Syndrome virus (PRRSv). Data are shown as individual S/p values, with error bars indicating the mean S/p value and standard deviation for each parity. Cut-off value for seropositivity (S/p ≥ 0.4) is shown as a dotted line. No significant differences in mean S/p values between different parities were found (*p* = 0.46). Despite routine PRRSv vaccination, 6/274 (parity 1), 12/296 (parity 2), 9/267 (parity 3) and 22/563 (parity 4+) sows were seronegative in ELISA 1. The proportion of seronegative sows was not significantly different between parities (*p* = 0.58).

In ELISA 1, 49 out of 1400 (3.5%; CI 95% [2.66, 4.60]) sows were identified as being PRRSv-seronegative. These 49 seronegative sows originated from 28/70 (40%) of the herds. The within-herd prevalence ranged from 5% to 20% (1 to 4 seronegative sows of 20 sampled). Next to the 49 seronegative sows, 84 sows (6.0%; CI 95% [4.87, 7.40]) were considered low-seropositive. Fifty out of seventy (71.43%) herds had at least one seronegative and/or one low-seropositive sow in ELISA 1. No significant differences (*p* = 0.58) in the proportion of seronegative sows between parities were observed, with the following proportions: parity 1, 6/274 (2.19%; CI 95% [1.01, 4.69]); parity 2, 12/296 (4.05%; CI 95% [2.33, 6.95]); parity 3, 9/267 (3.37%; CI 95% [1.78, 6.28]); and parity 4+, 22/563 (3.91%; CI 95% [2.59, 5.85]). Individual S/p values ranged from 0.04 (minimum) to 4.51 (maximum) with a mean S/p value for all sampled sows of 1.71 ± 0.83. Mean S/p values per herd ranged from 1.02 (minimum) to 2.94 (maximum). No significant differences (*p* = 0.46) in mean S/p values between different parities were observed (Figure 1).

### 3.4. Presence of ELISA 2 Non-Responding Sows

**Figure 2:**
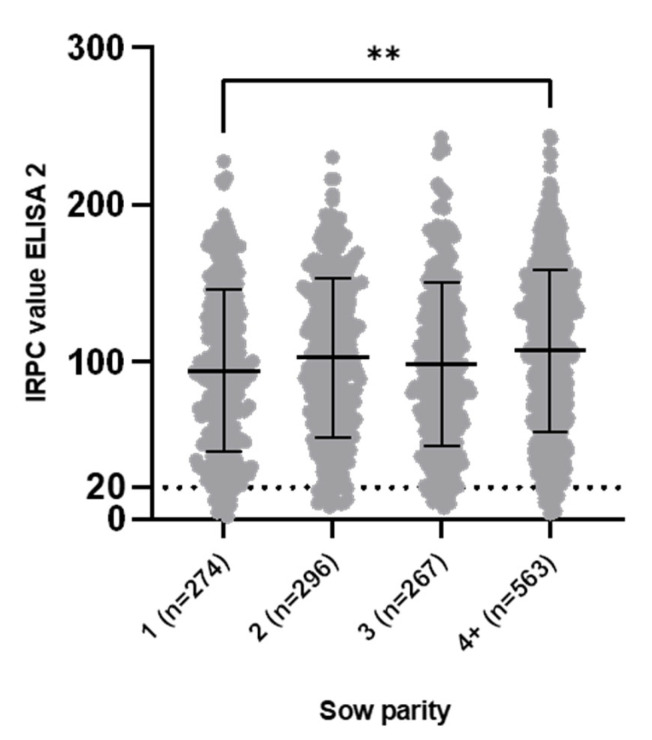
ELISA 2 (CIVTEST SUIS PRRS E/S) Relative Index Percent (IRPC) values in 1400 sow samples (parity 1 = 274, parity 2 = 296, parity 3 = 267 and parity 4+ = 563) originating from 70 Belgian sow herds that practice routine vaccination against Porcine Reproductive and Respiratory Syndrome virus (PRRSv). Data are shown as individual IRPC values, with error bars indicating the mean IRPC value and standard deviation for each parity. Cut-off value for seropositivity (IRPC > 20) is shown as a dotted line. A significant difference in mean IRPC value between parity 1 and parity 4+ sow was found (*p* = 0.004). Despite routine PRRSv vaccination, 14/274 (parity 1), 15/296 (parity 2), 11/267 (parity 3) and 18/563 (parity 4+) sows were seronegative in ELISA 2. The proportion of seronegative sows was not significantly different between parities (*p* = 0.47). ** *p* < 0.005.

In ELISA 2, a slightly higher proportion of sows tested seronegative: 58/1400 (4.14%; CI 95% [3.22%, 5.32%]). These 58 seronegative sows originated from 28/70 (40%) of the herds and the within-herd prevalence of ELISA 2 non-responding sows ranged from 5% to 30% (1 to 6 seronegative sows of 20 sampled). Next to the 58 seronegative sows, 70 sows (5%; CI 95% [3.98, 6.27]) were considered low-seropositive. Forty-four out of seventy (62.86%) herds had at least one seronegative or one low-seropositive sow in ELISA 2. No significant differences (*p* = 0.47) in the proportion of seronegative sows between different parities were observed, with the following proportions: parity 1, 14/274 (5.11%; CI 95% [3.07, 8.39]); parity 2, 15/296 (5.07%; CI 95% [3.10, 8.19]); parity 3, 11/267 (4.12%; 95% CI [2.32, 7.23]); and parity 4+, 18/563 (3.20%; CI 95% [2.03, 5.00]). Individual IRPC values ranged from 1.59 (minimum) to 243.84 (maximum) with a mean IRPC value for all sampled sows of 102.0 ± 51.69. The mean IRPC value per herd ranged from 33.73 (minimum) to 174.30 (maximum). A significant difference (d = −12.76; *p* = 0.004) in mean IRPC value between parity 1 and parity 4+ sows was observed. However, the biological significance of this difference is negligible. No significant differences were observed between the other parities (Figure 2). Importantly, 6/70 herds vaccinated their gilts and sows exclusively with a PRRSv-2 MLV. In theory, it would be expected that the sampled sows of these herds would lack ELISA-2-detectable Abs, since ELISA 2 is coated with a PRRSv-1 antigen. In contrast, ELISA 2 Abs were detected in 109/120 (90.83%) sows of these herds. The mean IRPC value of these six PRRSv-2-vaccinating herds (69.29 ± 43.48) was significantly lower (d = −35.79 ± 4.84; *p* < 0.0001) than the mean IRPC value of the 64 PRRSv-1-vaccinating herds (105.1 ± 51.35). Exclusion of the six PRRSv-2-vaccinating herds led to an adjusted proportion of 47/1280 (3.67%; CI 95% [2.77%, 4.85%]) seronegative sows in ELISA 2, which is more similar to the observed proportion of seronegative sows in ELISA 1.

### 3.5. Correlation between the ELISA 1 and ELISA 2 Results

**Figure 3:**
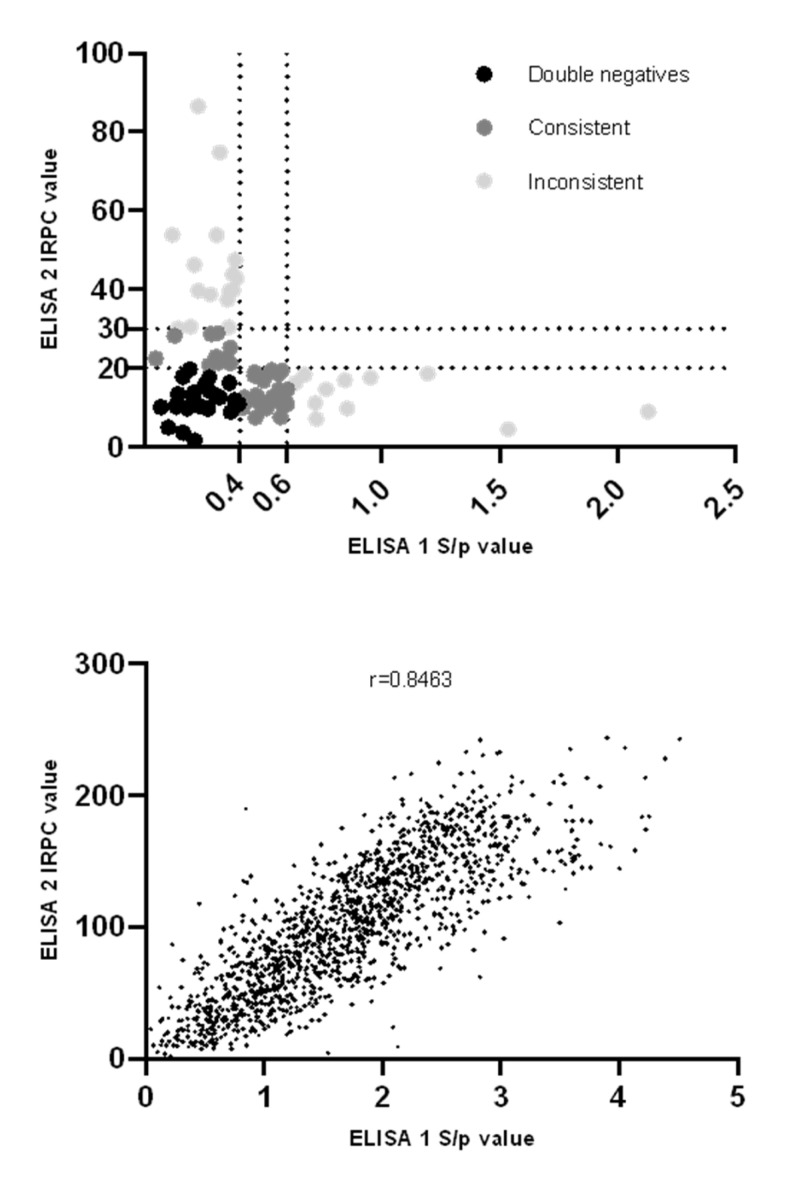
**(Above).** Overview of ELISA 1 (IDEXX PRRS X3) and ELISA 2 (CIVTEST SUIS PRRS E/S) results in the selection of 84 Porcine Reproductive and Respiratory Syndrome virus (PRRSv) vaccinated sows that tested seronegative in either or both ELISA tests. Individual sample-to-positive (S/p) (ELISA 1) and Relative Index Percent (IRPC) (ELISA 2) values for each sample are shown as dots. Dotted lines at S/p = 0.4 and IRPC = 20 show the cut-off values for seropositivity for ELISA 1 and ELISA 2, respectively. Dotted lines at S/p = 0.6 and IRPC = 30 show the cut-off values for low seropositivity for ELISA 1 and ELISA 2, respectively. Twenty-three sows were classified as being seronegative in both ELISA tests (double-negatives, black dots), 34 sows were classified as being seronegative in one ELISA test and low-seropositive in the other ELISA test (consistent results, dark grey), 27 sows were classified as being seronegative in one ELISA test and seropositive in the other ELISA test (inconsistent results, light grey). **(Below).** Overview of ELISA 1 (IDEXX PRRS X3) and ELISA 2 (CIVTEST SUIS PRRS E/S) results in 1400 sow samples originating from 70 Belgian sow herds that practice routine PRRSv vaccination. Individual sample-to-positive (S/p) (ELISA 1) and Relative Index Percent (IRPC) (ELISA 2) values for each sample are shown as dots. An overall Spearman correlation of r = 0.846 (CI 95% [0.830, 0.861], *p* < 0.0001) between ELISA 1 and ELISA 2 was found.

ELISA 1 and 2 results were combined, and 23 out of 1400 (1.64%; CI 95% [1.10, 2.45]) sows were identified as seronegative in both ELISA tests. These 23 double-seronegative (E1−/E2−) sows originated from 15/70 (21.4%) herds, with the within-herd prevalence ranging from 5% to 15% (1 to 3 E1−/E2− sows of 20 sampled). No significant differences (*p* = 0.44) in the proportion of E1−/E2− sows between different parities were observed, with the following proportions: parity 1, 4/274 (1.46%; CI 95% [0.57, 3.68]); parity 2, 8/296 (2.70%; CI 95% [1.38, 5.24]); parity 3, 3/267 (1.12%; CI 95% [0.31, 3.25]); and parity 4+, 8/563 (1.42%; CI 95% [0.72, 2.78]). Of the 26 ELISA 1 seronegative/ELISA 2 seropositive (E1−/E2+) sows, 10/26 (38.46%) were considered low-seropositive in ELISA 2. IRPC values of the remaining 16 E1−/E2+ sows ranged from 30.1 to 86.6, with a mean IRPC value of 45.96 ± 15.48. Of the 35 ELISA 1 seropositive/ELISA 2 seronegative (E1+/E2−) sows, 24/35 (68.57%) were considered low-seropositive in ELISA 1. S/p values of the remaining 11 E1+/E2− sows ranged from 0.64 to 2.13, with a mean S/p value of 1.00 ± 0.46. In summary (Figure 3a), 84/1400 (6.00% CI 95% [4.87, 7.37]) sows were identified as seronegative in either or both ELISA tests, originating from 37/70 (52.86%) of the herds. Of these 84 sows, 23/84 (27.38%) were seronegative in both ELISA tests, 34/84 (40.48%) were seronegative in one ELISA test and low-seropositive in the other ELISA test (consistent results) and 27/84 (32.14%) sows were seronegative in one ELISA test but seropositive in the other ELISA test (inconsistent results). A positive correlation between the ELISA 1 and 2 results was found (Figure 3b), with a Spearman correlation of r = 0.846 (CI 95% [0.830, 0.861], *p* < 0.0001). Exclusion of the six PRRSv-2-vaccinating herds did not alter the overall proportion of E1−/E2− sows: 21/1280 (1.64%; CI 95% [1.08, 2.50]) sows were seronegative in both ELISA tests. Exclusion resulted in a slightly better correlation between ELISA 1 and ELISA 2 results, with a Spearman correlation of r = 0.855 (CI 95% [0.839, 0.869], *p* < 0.0001).

### 3.6. Confirmation of ELISA 1 and/or ELISA 2 Non-Responders in ELISA 3 and ELISA 4

Seronegative samples in either ELISA 1 and/or ELISA 2 were further analyzed using two additional ELISA kits (Table 3) to confirm their seronegative status. Of the 23 E1−/E2− sows, 22/23 (95.65%) tested seronegative in ELISA 3, with one seropositive sample just above the cut-off value: S/p value = 0.41. In ELISA 4, 21/23 (91.3%) E1−/E2− sows were classified as seronegative, with two seropositive samples having S/p values of 0.75 and 1.08. The E1−/E2+ sows mostly tested seronegative in the additional kits as well: 25/26 (96.15%) were seronegative in ELISA 3, and 24/26 (92.31%) tested seronegative in ELISA 4. S/p values of the seropositive samples were 1.44 for the ELISA 3 seropositive sample and 0.54 and 1.35 for the ELISA 4 seropositive samples. Finally, for the E1+/E2− samples, 30/35 (85.71%) tested seronegative in ELISA 3, while 27/35 (77.14%) were seronegative in ELISA 4. S/p values of the ELISA 3 seropositive samples ranged from 0.52 to 1.54, and S/p values of the ELISA 4 seropositive samples ranged from 0.57 to 1.06.

### 3.7. Results of the Virus Neutralization Assay (VN)

A selection of 319 samples was made for VN: 81 ELISA seronegative samples (23 E1−/E2− samples, 26 E1−/E2+ samples and 32 E1+/E2− samples; lack of serum for 3 E1+/E2− samples), 76 ELISA low-seropositive samples (S/p ELISA 1: {0.4, 0.6} and/or IRPC ELISA 2: {20, 30}) and 162 ELISA seropositive samples (S/p ELISA 1: {0.6, 3.55} and IRPC ELISA 2: {31,07, 232.5}), with at least two seropositive samples of each herd. Results of the VN provide additional information concerning the humoral immune status of the routinely vaccinated sows: it is well known that PRRSv-specific ELISA Abs are not necessarily correlated to protection, in contrast to the PRRSv-specific NAbs that have been shown to protect against clinical disease and infection [28].

**Figure 4:**
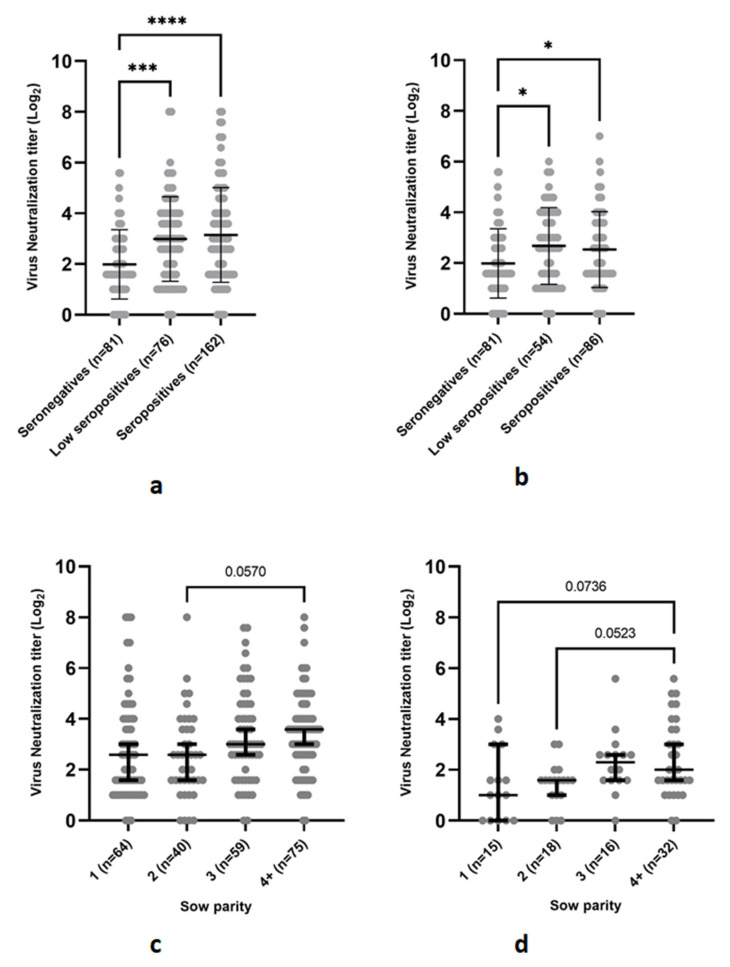
Quantification of neutralizing antibodies (NAbs) against the Porcine Reproductive and Respiratory Syndrome virus (PRRSv)-1 DV strain by means of a virus neutralization assay (VN) in a selection of 319 sow samples, originating from 70 Belgian sow herds that practice routine PRRSv vaccination. (**a**) Comparison of VN titers between ELISA seronegative, low-seropositive and seropositive samples in the selection of 319 sow samples. Individual VN titers (Log_2_) are shown for a selection of ELISA seronegative (*n* = 81), ELISA low-seropositive (*n* = 76) and ELISA seropositive (*n* = 162) sows. Error bars indicate the mean VN titer (Log_2_) and standard deviation. ELISA seronegative sows have significantly less NAbs compared to the ELISA low-seropositive (*p* = 0.0005) and ELISA seropositive sows (*p* < 0.0001). Cut-off value for seropositivity is shown as a dotted line. (**b**) Comparison of VN titers between ELISA seronegative, low-seropositive and seropositive samples in a selection of 221 samples belonging to 37 different herds (exclusion of 33 herds without any ELISA seronegative sows). Individual VN titers (Log_2_) are shown for a selection of seronegative (*n* = 81), low-seropositive (*n* = 54) and seropositive (*n* = 86) sows. Error bars indicate mean VN titer (Log_2_) and standard deviation. ELISA seronegative sows have significantly less NAbs compared to the ELISA low-seropositive (*p* = 0.022) and ELISA seropositive sows (p = 0.043). Cut-off value for seropositivity is shown as a dotted line. (**c**) Comparison of VN titers between parity 1, 2, 3 and 4+ sows in a selection of 238 ELISA seropositive samples. Individual VN titers (Log_2_) are shown for a selection of parity 1 (*n* = 64), parity 2 (*n* = 40), parity 3 (*n* = 59) and parity 4+ (*n* = 75) ELISA seropositive sows. Error bars indicate mean SN titer (Log_2_) and standard deviation for each parity. No significant differences in mean VN titers of parity 1 (2.83 ± 2.01 Log_2_), parity 2 (2.54 ± 1.69 Log_2_), parity 3 (3.34 ± 1.80 Log_2_) and parity 4+ (3.43 ± 1.6 Log_2_) sows were found. Cut-off value for seropositivity is shown as a dotted line. (**d**) Comparison of VN titers between parity 1, 2, 3 and 4+ sows in a selection of 81 ELISA seronegative samples. Individual VN titers (Log_2_) are shown for a selection of parity 1 (*n* = 15), parity 2 (*n* = 18), parity 3 (*n* = 16) and parity 4+ (*n* = 32) ELISA seronegative sows. Error bars indicate mean VN titer (Log_2_) and standard deviation for each parity. No significant differences in mean VN titers of parity 1 (1.42 ± 1.39 Log_2_), parity 2 (1.43 ± 0.86 Log_2_), parity 3 (2.28 ± 1.23 Log_2_) and parity 4+ (2.43 ± 1.5 Log_2_) sows were found. Cut-off value for seropositivity is shown as a dotted line. * *p* < 0.05, *** *p* < 0.0005, **** *p* < 0.0001.

The mean VN titer of the ELISA seronegative samples was negative (1.99 ± 1.37 Log_2_). However, there was a quite high proportion of ELISA seronegative samples that tested positive in VN (35/81; 43.3%). In contrast, the mean VN titers of both the ELISA low-seropositive samples (2.99 ± 1.67 Log_2_) and ELISA seropositive samples (3.15 ± 1.87) were positive. Significant differences in VN titer were found between the ELISA seronegative samples and ELISA low-seropositive samples (*p* = 0.005) and between the ELISA seronegative samples and ELISA seropositive samples (*p* < 0.0001) but not between the ELISA low-seropositive samples and ELISA seropositive samples (*p* = 0.50) (Figure 4a). Furthermore, the ELISA (low) seropositive samples had a significantly higher proportion of VN positives (167/238; 70.2%) compared to the ELISA seronegative samples (*p* < 0.0001). Exclusion of (low) seropositive samples originating from herds without any ELISA 1 nor ELISA 2 seronegative samples led to a similar conclusion (Figure 4b): VN titers of the 81 ELISA seronegative samples (1.99 ± 1.37 Log_2_) were significantly lower than the VN titers of the 54 remaining, low-seropositive samples (*p* = 0.02, 2.68 ± 1.51 Log_2_) and the VN titers of the 86 remaining seropositive samples (*p* = 0.043, 2.54 ± 1.50 Log_2_). ELISA seronegativity in either ELISA 1, ELISA 2 or both ELISA 1 and ELISA 2 did not influence the presence of NAbs, with no significant differences (*p* = 0.87) found between the VN titers of the 23 E1−/E2− (1.92 ± 1.03 Log_2_), 26 E1−/E2+ (2.11 ± 1.45 Log_2_) and 32 E1+/E2− (1.94 ± 1.54 Log_2_) samples (Figure S2). The selection of 319 samples contained forty PRRSv-2-vaccinated sows, derived from the six PRRSv-2-vaccinating herds. The mean VN titer of these 40 PRRSv-2-vaccinated sows was 2.16 ± 1.35 Log_2_, and 19/40 (47.5%) of these sows were considered seropositive in VN, with VN titers ≥ 2 Log_2_.

Finally, the influence of sow parity on the observed VN titers was further investigated. In the selection of ELISA (low) seropositive sows (*n* = 238), the largest difference (d = −0.89; *p* = 0.057) was observed between VN titers of parity 2 (2.54 ± 1.69 Log_2_) and parity 4+ (3.43 ± 1.60 Log_2_) sows (Figure 4c). In the selection of ELISA seronegative sows (*n* = 81), a large difference (d = −1.01; *p* = 0.07) was observed between parity 1 (1.42 ± 1.34 Log_2_) and parity 4+ (2.43 ± 1.5 Log_2_) sows. Additionally, a large difference (d = −1; *p* = 0.052) was found between parity 2 (1.43 ± 0.86 Log_2_) and parity 4+ sows (Figure 4d).

### 3.8. Herd Risk Factor Analysis

The time between last PRRSv vaccination and date of sampling was significantly longer in seronegative herds compared to seropositive herds. No significant differences in biosecurity scores were observed between seropositive and seronegative herds. Seronegative herds had a slightly higher number of sows, but this difference was not significant (Table 4). No specific MLV vaccine was identified as a possible risk factor for the presence of ELISA non-responders, with approximately 50% of the herds using either Ingelvac MLV, Porcilis or Unistrain, having at least one ELISA non-responder. None of the herds (0/16) using the combination of one MLV (Porcilis) and one KV (Progressis) had ELISA non-responders, suggesting that this combination strongly reduces the risk of PRRSv seronegativity (OR = 0; CI 95% [0, 0.4]; *p*-value = 0.006). The latter is also reflected in the sow PRRSv vaccination scheme used: a significantly reduced risk (OR = 0.1; CI 95% [0, 0.4]; *p*-value = 0.014) was observed in herds vaccinating their sows at 60 and 90 days of gestation, compared to the other vaccination schemes. This scheme was exclusively used in herds vaccinating the sows with an MLV (60 days of gestation) and a KV (90 days of gestation). Herd variables related to gilt management were not significantly related to the presence of ELISA non-responding sows. Finally, the used administration route (intramuscular vs. intradermal) was not identified as a possible risk factor for non-responsiveness (Table 5).

## 4. Discussion

The results of this cross-sectional study showed a relatively low (3.5–4.1%) presence of multiple, PRRSv-vaccinated but ELISA seronegative (ELISA non-responding) sows in Belgium. Despite the low global prevalence, the found non-responders were widely distributed, with 40% of the sampled herds having at least one non-responding sow on twenty sows sampled. A first indication of the relevance of the ELISA non-responders was given by the results of the VN: the ELISA non-responders had significantly less NAbs compared to the ELISA responders. Finally, the risk of having non-responding sows was significantly reduced when the combination of one MLV (at 60 days of gestation) and one KV (at 90 days of gestation) was used.

Seventy Belgian sow herds, originating from six different provinces/regions, were included in this cross-sectional study. All selected herds practiced routine PRRSv vaccination of their sow population. Nevertheless, 36% of the herd owners still reported having had problems related to PRRS in the year preceding the sampling date, confirming that the effectiveness of PRRSv vaccination remains suboptimal in some cases [8,10]. Results of the PRRSv-specific questionnaire showed a need for better guidelines concerning PRRSv vaccination in Belgium. First, a lot of variation was seen in the PRRSv vaccine schedule used: ten different PRRSv vaccines/PRRSv vaccine combinations were administered and twelve different PRRSv vaccination schemes were practiced. Second, despite the possible risk for recombination between different MLV strains [12,13,14,15,16,17,18], four herds vaccinated their sows with a combination of two different MLVs, six herds vaccinated their gilts with a combination of two different MLVs and five herds vaccinated their gilt and sow population with different MLVs. Finally, six herds exclusively vaccinated their gilt and sow population with a PRRSv-2 MLV, despite the fact that PRRSv-1 strains are more dominant in Belgium. Next to proper PRRSv vaccination, adequate biosecurity practices can aid in prevention of clinical disease. Biosecurity scores, as well as observations, during the herd visits showed a lot of variation between herds and made clear that biosecurity could still be significantly improved in some herds.

All 1400 samples were tested on two commercially available ELISA kits: ELISA 1 and ELISA 2. An overall strong positive correlation was found between the ELISA 1 and ELISA 2 results. However, upon closer inspection of the 84 ELISA seronegative samples (in either or both tests), some discrepancies in classification were observed. Additionally, the 84 ELISA seronegative samples (in either or both tests) were further analyzed on two other ELISA kits: ELISA 3 and 4. Although most of the ELISA 1 and/or ELISA 2 sows were also seronegative in ELISA 3 and 4, some discrepancies in classification were again observed. The discrepancies can be explained by differences in both specificity and sensitivity between different ELISA tests [29,30]. Biernacka et al. [29] reported a specificity of 100% for both ELISA 1 and ELISA 2 and a sensitivity of 80.3% for ELISA 2 relative to the sensitivity of ELISA 1. Furthermore, ELISA 2 is coated with a different antigen than ELISA 1, which could further explain the differences in classification between both tests. Interestingly, six herds exclusively vaccinated their gilt and sow population with a PRRSv-2 MLV, but a large proportion (90.8%) of the sows originating from these herds had ELISA-2-detectable Abs. This suggests that there is either a PRRSv-1 strain circulating in these herds, causing the production of ELISA-2-detectable Abs or, alternatively, the elicited PRRSv-2 Abs cross-react with the PRRSv-1 antigen of ELISA 2. The latter was also suggested by Sattler et al. [25], in which a proportion of pigs experimentally infected with a PRRSv-2 MLV tested seropositive in ELISA 2.

To obtain a first indication of the relevance of the ELISA non-responders, a VN was performed to determine the presence of PRRSv-specific NAbs. VN was performed against the PRRSv-1 DV strain, the PRRSv strain from which the Porcilis vaccine is derived, the vaccine which was most frequently used in the selected herds. Firstly, VN results showed no meaningful differences between ELISA low seropositive and ELISA seropositive samples, with both groups having high amounts of NAbs and a high proportion of VN positives. This suggests that the observed ELISA low seropositivity is not due to an inadequate response to PRRSv-vaccination but rather the result of pre-existing immunity which might inhibit the humoral boost after vaccination. Secondly, the VN results showed that the ELISA non-responding sows (in either or both ELISA 1 and 2) had significantly lower NAbs compared to the ELISA (low) seropositive sows, with the latter having twofold higher neutralizing titers compared to the ELISA non-responders. Furthermore, a significant difference in the proportion of VN seropositive samples between the ELISA (low) seropositive and ELISA seronegative samples was observed.

Based on these results, a first indication of the possible relevance of the ELISA non-responding sows was provided, especially in the proportion of ELISA non-responders that are also negative in VN. However, additional investigation, with a particular focus on the cell-mediated immune responses (CMI), is needed to further explore the immune status of the ELISA non-responders. Finally, the combination of both ELISA and VN might provide a better tool for determining the (humoral) immune status of multiple PRRS-vaccinated sows, but this is less feasible in practice. In this study, an adjusted proportion of 46/1400 (3.3%) non-responders was found when both ELISA and VN are taken into account: 46 multiple PRRSv-vaccinated sows tested negative in at least one ELISA kit and in VN. Unfortunately, VN was not carried out against another PRRSv-1 strain, due to a lack of collected serum.

A secondary aim of this cross-sectional study was to identify herd risk factors for the presence of ELISA non-responders. Due to the low frequency of ELISA seronegative samples, the large variety in herd variables and the presence of one herd variable with perfect separation, namely the used PRRSv vaccine (combination Porcilis + Progressis), it was not feasible to build a representative multivariable herd model. For this reason, herd risk factors were only analyzed in a univariable way.

A strongly reduced risk for the presence of ELISA 1 non-responding sows was found in herds that vaccinate their sows at 60 days of gestation with an MLV and at 90 days of gestation with a KV. Three possible explanations for this reduced risk could be hypothesized. First, ELISA non-responsiveness might arise due to improper vaccination practices, e.g., mistakes in administration route, dosage, etc. By administering two vaccines at two different time points, the risk of improper vaccination is strongly reduced. Second, non-responsiveness might arise from a repeated vaccination effect: routine vaccination with the same MLV might eventually loses the capability of eliciting an adequate immune response [31,32]. This repeat effect might be counteracted by the administration of a KV (different strain as the MLV). Third, the ELISA non-responding sow might have an initial, weak, response to vaccination, followed by a rapid drop in Ab levels, causing the non-responding sow to become ELISA seronegative. Since most of the sows in this study were sampled in the farrowing unit, the time since KV vaccination (90 days of gestation) and sampling is rather short; thus, non-responding sows with a rapid drop in Abs could still test seropositive in ELISA. The latter is further strengthened by the observation that the time since last PRRSv vaccination and moment of sampling was significantly longer in herds having the presence of at least one ELISA 1 seronegative sow (of 20 sampled) compared to herds without ELISA 1 non-responding sows. On the one hand, this seems like a logical observation. However, in 95% of the sampled herds, the time between last PRRSv vaccination and sampling was no longer than 4 months (two herds: 5 months/one herd: 6 months). Since Abs against the N protein normally persist for more than 4 months (24-32 weeks post-vaccination/infection [33,34]), the time between last PRRSv vaccination and moment of sampling should not be an issue for detecting ELISA 1 non-responders in this sample population, unless some sows elicit a more rapid drop of PRRSv-specific Abs.

No other herd risk factors for the presence of ELISA non-responding sows were found. The used MLV strain, which could be hypothesized to be an important determining factor, did not increase nor decrease the risk of having ELISA 1 non-responders. Another possible factor, the administration route (IM vs. ID), did not influence the presence of ELISA 1 non-responders in this study.

No significant influence of sow parity on the observed ELISA non-responsiveness was found. However, parity 2 sows had a slightly higher proportion of ELISA 1 and ELISA 2 seronegative samples compared to the other parities. Furthermore, parity 2 sows had less NAbs, with p-values just above the cut-off for significance, compared to parity 4+ sows. These two findings suggest that parity 2 sows might react less to PRRSv vaccination compared to the other parities. Two possible explanations could be proposed for this hypothesis. First, PRRSv immunity might simply increase with increasing parity: the older the sow, the more PRRSv vaccinations (and wild virus interactions) she has received and, consequently, the higher the PRRSv immune status becomes. Parity 1 sows could be considered a distinct group in this hypothesis, since primiparous sows are generally intensively vaccinated in both the origin herd (in case of purchase) and quarantine unit, resulting in a high immune status before entering the sow stable [35]. Alternatively, one could hypothesize that the intensive PRRSv vaccination in primiparous sows could lead to a temporary non-responsiveness to PRRSv vaccination, resulting in a drop in immunity in parity 2 sows. Additional studies are needed to explore these hypotheses and to further elicit the influence of sow parity on PRRSv immunity.

This study, aimed at detecting the presence of PRRSv-vaccinated, ELISA non-responding sows had some clear strengths as well as some limitations. The study was based on a large sampling, covering more than 5% of Belgian sow herds, and was representative for the geographic distribution and herd size of Belgian sow herds. The median number of sows present on the herds was 305, but outliers (in both directions) were included, avoiding a certain herd-size effect: eleven herds had only 200 or less sows on site, while sixteen herds had 500 or more sows on site. In each herd, sows of different parities were sampled, enabling the investigation of parity influence on the observed PRRSv immune responses. All herd visits were carried out by the PI, allowing the PI to verify the answers given on the questionnaires and to ensure that all blood samples were correctly identified (herd, sow and parity). To avoid a certain ELISA kit effect on the observed results, multiple ELISAs were used to determine the immune status of the sampled sows. The cross-sectional design allowed us to have a snapshot of the real field situation at a given time, but this design has some limitations as well. One obvious limitation was the lack of humoral follow-up: ELISA non-responders were not followed-up during this study to investigate whether or not they seroconvert after the next PRRSv vaccination. Furthermore, there was no control over the correct administration of PRRSv vaccination. Finally, selection of herds was not completely at random: the respective herd veterinarians selected herds that were interested to participate in this study. To avoid selection bias as much as possible, different veterinarians participated in this study.

## 5. Conclusions

The overall proportion of ELISA non-responders was relatively low (3.5–4.1%), but the proportion of herds having ELISA non-responders was quite high (40%), suggesting that this phenomenon is not limited to specific herds. Several hypotheses that might explain the origin of non-responders arose from this study, but additional research is needed to explore these. A first indication of the biological significance of the ELISA non-responders was provided by the fact that ELISA non-responding sows had significantly less NAbs compared to the ELISA responding sows. However, next to the humoral immune responses, cell-mediated immunity (CMI) plays an important role in the protection against PRRSv infection. CMI was not investigated in the current study and could provide a more complete image of the PRRSv immune status of the ELISA non-responders. Finally, it could be hypothesized that piglets born from non-responding sows might lack maternally derived immunity and are consequently less protected against PRRSv infection. Additionally, these piglets might react differently to PRRSv vaccination themselves compared to piglets born from responding sows. The possible consequences of this peculiar sow PRRSv immune status for the progeny warrant further investigation.

## Figures and Tables

**Table 1:** Biosecurity data of 70 selected Belgian sow herds that practice routine vaccination against Porcine Reproductive and Respiratory Syndrome virus (PRRSv). Biosecurity scores are determined by the BioCheck questionnaire. Scores are shown as the mean ± standard deviation (SD) and minimum and maximum score for each biosecurity category. Scores can range from a minimum of 0 to a maximum of 100.

Biosecurity Category	Mean Score ± SD	Minimum Score	Maximum Score
External biosecurity	71.5 ± 8.5	53	91
Purchase of breeding pigs, piglets and semen	84.6 ± 11.5	58	100
Transport of animals, removal of carcasses and manure	76.5 ± 12.4	48	100
Feed, water and equipment supply	49.3 ± 15.3	17	100
Visitors and farmworkers	73.5 ± 17.7	35	100
Vermin and bird control	78.6 ± 18.8	30	100
Location of the farm	49.7 ± 21.3	0	100
Internal biosecurity	63.4 ± 13.5	35	90
Disease management	72.6 ± 24.8	20	100
Farrowing and suckling period	52.8 ± 18.9	14	86
Nursery unit ^1^	75.3 ± 15.9	36	100
Finishing unit ^2^	81.6 ± 19.7	21	100
Measures between compartments, working lines and use of equipment	50.3 ± 22.2	11	100
Cleaning and disinfection	66.8 ± 23.5	0	100
Total biosecurity	67.7 ± 10.1	48	87

^1^*n* = 69, exclusion of 1 herd without nursery unit; ^2^ *n* = 52, exclusion of 18 herds without finishing unit.

**Table 2:** Categorical data related to sow, gilt and piglet vaccination management in the selection of 70 Belgian sow herds that practice routine vaccination against Porcine Reproductive and Respiratory Syndrome virus (PRRSv). Data were gathered by means of a PRRSv-specific questionnaire and were applicable to each farm for the period of 12 months prior to the farm visit. Data are shown as the number (*n*) and percentage (%) of herds having a certain herd variable.

Herd Variable	*n* Herds	% Herds
Used PRRSv vaccine in sows	70	
Porcilis (PRRSv-1 MLV)	27	38.6
Porcilis (PRRSv-1 MLV) + Progressis (PRRSv-1 KV)	16	22.9
Unistrain (PRRSv-1 MLV)	12	17.1
Ingelvac MLV (PRRSv-2 MLV)	6	8.6
Ingelvac MLV (PRRSv-2 MLV) + Porcilis (PRRSv-1 MLV)	3	4.3
Progressis (PRRSv-1 KV)	2	2.9
Ingelvac PRRSFlex (PRRSv-1 MLV)	1	1.4
Reprocyc (PRRSv-1 MLV)	1	1.4
Ingelvac MLV (PRRSv-2 MLV) + Unistrain (PRRSv-1 MLV)	1	1.4
Ingelvac MLV (PRRSv-2 MLV) + Progressis (PRRSv-1 KV)	1	1.4
Route of PRRSv vaccine administration in sows ^1^	70	
Intramuscular (IM)	40	57.1
Intradermal (ID)	30	42.9
Used PRRSv vaccination scheme in sows	70	
Group: every 4 months	21	30.0
Group: every 3 months	14	20.0
60 days + 90 days of gestation	13	18.6
60 days gestation + 6 days post-farrowing	9	12.9
Group: every 3.5 months	3	4.3
60 days of gestation + 15 days post-farrowing	2	2.9
In the farrowing unit	2	2.9
90 days of gestation + 14 days post-farrowing	2	2.9
Group: every 3 months + 90 days of gestation	1	1.4
90 days of gestation + 6 days post-farrowing	1	1.4
42 days of gestation	1	1.4
60 days of gestation	1	1.4
Time between blood sampling and last PRRSv vaccination in sows	70	
<1 month	8	11.4
1 month	20	28.6
1.5 months	5	7.1
2 months	16	22.9
2.5 months	1	1.4
3 months	11	15.7
3.5 months	2	2.9
4 months	4	5.7
5 months	2	2.9
6 months	1	1.4
Other vaccines used in sows	70	
Parvovirus + *Erysipelothrix rhusiopathiae*	67	95.7
*Escherichia coli*	58	82.9
Atrophic rhinitis	54	77.1
Influenza virus	39	55.7
*Clostridium perfringens*	36	51.4
*Glässerella parasuis*	22	31.4
Rotavirus	20	28.6
*Actinobacillus pleuropneumoniae*	12	17.1
Porcine Circovirus type 2	11	15.7
*Mycoplasma hyopneumoniae*	7	10
Clinical problems with PRRSv in sows	70	
Yes	25	32.7
No	45	64.3
Clinical signs of PRRSv in sows	25	
Abortions	18	72.0
Premature birth	16	64.0
Stillborn/weak born piglets	10	40.0
Increased mortality in farrowing unit	6	24.0
Other	4	16.0
Used PRRSv vaccine in gilts ^2^	69	
Porcilis (PRRSv-1 MLV)	31	44.9
Unistrain (PRRSv-1 MLV)	13	18.8
Ingelvac MLV (PRRSv-2 MLV)	10	14.5
Ingelvac MLV (PRRSv-2 MLV) + Porcilis (PRRSv-1 MLV)	5	7.3
Progressis (PRRSv-1 KV)	4	5.8
Porcilis (PRRSv-1 MLV) + Progressis (PRRSv-1 KV)	3	4.4
Ingelvac MLV (PRRSv-2 MLV) + Unistrain (PRRSv-1 MLV)	1	1.5
Ingelvac PRRSFlex (PRRSv-1 MLV) + Progressis (PRRSv-1 KV)	1	1.5
Reprocyc (PRRSv-1 MLV) + Progressis (PRRSv-1 KV)	1	1.5
Route of PRRSv vaccine administration in gilts ^2^	69	
Intramuscular (IM)	53	76.8
Intradermal (ID)	16	23.2
Purchasing of gilts	70	
Yes	46	65.7
No	24	34.3
PRRSv vaccination of gilts at origin herd	46	
Yes	38	82.6
No	8	17.4
Other vaccines used in gilts	70	
Parvovirus + *Erysipelothrix rhusiopathiae*	66	94.3
Influenza virus	43	61.4
Porcine Circovirus type 2	34	48.6
*Mycoplasma hyopneumoniae*	34	48.6
Atrophic rhinitis	30	42.9
*Actinobacillus pleuropneumoniae*	24	34.3
*Glässerella parasuis*	24	34.3
*Escherichia coli*	23	32.9
*Clostridium perfringens*	15	21.4
Rotavirus	7	10.0
PRRSv vaccination of piglets	70	
Yes	32	45.7
No	38	54.3
Used PRRSv vaccine in piglets	32	
Porcilis (PRRSv-1 MLV)	12	37.5
Suvaxyn MLV (PRRSv-1 MLV)	9	28.1
Unistrain (PRRSv-1 MLV)	4	12.5
Ingelvac PRRSFlex (PRRSv-1 MLV)	4	12.5
Ingelvac MLV (PRRSv-2 MLV)	3	9.4
Route of PRRSv vaccine administration in piglets	32	
Intramuscular (IM)	21	65.6
Intradermal (ID)	11	34.4
Clinical problems with PRRSv in piglets	70	
Yes	26	37.1
No	44	62.9
Clinical signs of PRRSv in piglets	26	
Coughing	18	69.2
Sneezing	12	46.2
Increased mortality in nursery unit	11	42.3
Dyspnea	6	23.1
Other	5	19.2

^1^ In case of use of two PRRSv vaccines: if one is administered ID, the herd is classified as ID; ^2^ gilts are not PRRSv vaccinated in one herd (arrive in gestation).

**Table 3:** Additional analysis of 84 ELISA 1 (IDEXX PRRS X3) and/or or ELISA 2 (CIVTEST SUIS PRRS E/S) seronegative sows on ELISA 3 (INgezim PRRS 2.0) and ELISA 4 (ID Screen PRRS Indirect). Number (%) of ELISA 3 and ELISA 4 seronegative samples in the selection of 84 ELISA 1 and/or ELISA 2 seronegative samples. Samples with a sample-to-positive ratio <0.4 were considered to be seronegative in ELISA 3 and 4.

	*n*	ELISA 3 Seronegative Samples (%)	ELISA 4 Seronegative Samples (%)
**ELISA 1 (−)/ELISA 2 (−)**	23	22 (95.7)	21 (91.3)
**ELISA 1 (−)/ELISA 2 (+)**	26	25 (96.1)	24 (92.3)
**ELISA 1 (+)/ELISA 2 (−)**	35	30 (85.7)	27 (77.1)

**Table 4:** Unpaired t-test/Mann–Whitney U test comparing the differences in mean/median values of continuous variables between “seronegative” (herds with at least one ELISA 1 (IDEXX PRRS X3) seronegative sow) and “seropositive” (herds without any ELISA 1 seronegative sow) herds. Results are shown as the mean ± standard deviation (SD) or the median ± interquartile range (IQR) value for the continuous variable in the seropositive (*n* = 42) and seronegative (*n* = 28) herds. Differences with a p-value < 0.05 are considered to be significant.

Continuous Variable	Seropositive Herds (*n* = 42) Mean ± SD	Seronegative Herds (*n* = 28) Mean ± SD	*p*-Value
External biosecurity	70.9 ± 7.5	72.4 ± 9.8	0.47
Purchase of breeding pigs, piglets and semen	83.71 ± 11.4	86 ± 11.7	0.42
Transport of animals, removal of carcasses and manure	76.3 ± 12.3	76.8 ± 12.9	0.88
Feed, water and equipment supply	48.9 ± 14.0	50.0 ± 17.4	0.77
Visitors and farmworkers	72.2 ± 14.9	75.3 ± 14.5	0.40
Vermin and bird control	78.8 ± 18.1	78.2 ± 20.2	0.90
Location of the farm	48.8 ± 21.1	51.1 ± 21.8	0.67
Internal biosecurity	62.1 ± 13.8	65.3 ± 13.2	0.35
Disease management	70.5 ± 23.9	75.7 ± 26.3	0.39
Farrowing and suckling period	50.1 ± 20.1	56.9 ± 16.6	0.14
Nursery unit ^1^	74.7 ± 17.5	76.2 ± 13.6	0.70
Finishing unit ^2^	81.7 ± 20.6	81.5 ± 18.8	0.98
Measures between compartments, working lines and use of equipment	48.1 ± 20.4	53.7 ± 24.5	0.30
Cleaning and disinfection	68.7 ± 23.6	63.9 ± 23.6	0.41
Total biosecurity	66.8 ± 9.6	69.1 ± 10.8	0.36
Time between last PRRSv vaccination and sampling (months)	1.6 ± 0.9	2.5 ± 1.4	0.002
Herd size (number of sows) ^3^	300 ± 192.5	324 ± 200	0.14

^1^*n* = 69, exclusion of 1 herd without nursery unit; ^2^ *n* = 52, exclusion of 18 herds without finishing unit; ^3^ result shown as median ± IQR; Mann–Whitney U test.

**Table 5:** Univariable logistic regression of the categorical herd variables ~ presence of at least one ELISA 1 (IDEXX PRRS X3) seronegative sow (“seronegative herd”). Results are shown as the number (*n*) of seropositive and seronegative herds for each variable, with the percentage of seropositive and seronegative herds for each variable between brackets. The odds ratio (OR) and 95% confidence interval (CI 95%) of the OR express the risk for being a seronegative herd when having a certain herd variable, compared to the reference variable. OR with a p-value < 0.05 is considered significant.

Variable	*n* (%) Seropositive Herds	*n* (%) Seronegative Herds	OR [CI 95%]	*p*-Value
Used PRRSv vaccine in sows				
Ingelvac PRRS MLV	3 (50%)	3 (50%)	Reference	Reference
Porcilis + Progressis	16 (100%)	0 (0%)	0 [0, 0.4]	0.006
Unistrain	6 (50%)	6 (50%)	1 [0.2, 6.6]	1
Porcilis	12 (44.4%)	15 (55.6%)	1.2 [0.2, 6.9]	0.799
Other vaccine/vaccine combinations	5 (55.6%)	4 (44.4%)	0.8 [0.1, 5.9]	0.839
Route of PRRSv vaccine administration in sows ^1^				
Intra-muscular (IM)	24 (60%)	16 (40%)	Reference	Reference
Intra-dermal (ID)	18 (60%)	12 (40%)	1 [0.4, 2.6]	1
Used PRRSv vaccination scheme in sows				
Group: each 4 months	9 (42.9%)	12 (57.1%)	Reference	Reference
60 days + 90 days of gestation	12 (92.3%)	1 (7.7%)	0.1 [0, 0.4]	0.014
Group: each 3 months	9 (64.3%)	5 (35.7%)	0.4 [0.1, 1.6]	0.218
60 days gestation + 6 days post-farrowing	4 (44.4%)	5 (55.6%)	0.9 [0.2, 4.8]	0.936
Other scheme	8 (61.5%)	5 (38.5%)	0.5 [0.1, 1.9]	0.293
Used PRRSv vaccine in gilts				
Ingelvac PRRS MLV	5 (50%)	5 (50%)	Reference	Reference
Porcilis + Progressis	3 (100%)	0 (0%)	0.1 [0, 2]	0.165
Unistrain	6 (46.2%)	7 (53.8%)	1.2 [0.2, 5.8]	0.859
Porcilis	21 (67.7%)	10 (32.3%)	0.5 [0.1, 2]	0.314
Other vaccine/vaccine combinations	6 (50%)	6 (50%)	1 [0.2, 5.1]	1
Route of PRRSv vaccine administration in gilts ^1^				
Intra-muscular (IM)	31 (58.5%)	22 (41.5%)	Reference	Reference
Intra-dermal (ID)	10 (62.5%)	6 (37.5%)	0.8 [0.3, 2.6]	0.775
Purchasing of gilts				
No	13 (54.2%)	11 (45.8%)	Reference	Reference
Yes	29 (63%)	17 (37%)	0.7 [0.3, 1.9]	0.473

^1^ In case of use of two PRRSv vaccines: if one is administered ID, the herd is classified as ID.

## Data Availability

The data presented in this study are available on request from the corresponding author. The data are not publicly available due to privacy reasons.

## Supplementary Materials

The following supporting information can be downloaded at: https://www.mdpi.com/article/10.3390/v14091944/s1, Figure S1: Geographic distribution of the study’s sow farms (*n* = 70) compared to the geographic distribution of Belgian sow farms (*n* = 1099). Figure S2: Quantification of neutralizing antibodies (NAbs) against the Porcine Reproductive and Respiratory Syndrome virus (PRRSv)-1 DV strain by means of a Virus Neutralization assay (VN) in a selection of 81 ELISA 1 (IDEXX PRRS X3) and/or ELISA 2 (CIVTEST SUIS PRRS E/S) seronegative sows, originating from 37 Belgian sow herds that practice routine PRRSv vaccination.
