# Supplementary material for: Porcine Reproductive and Respiratory Syndrome virus (PRRSv): A Cross-Sectional Study on ELISA Seronegative, Multivaccinated Sows
Source: Viruses. 2022 Aug 31;14(9):1944. doi: 10.3390/v14091944 (PMC9501492; doi:10.3390/v14091944)
Supplement: Supplementary file 1 [file viruses-14-01944-s001.zip › Supplementary Figures.pdf]

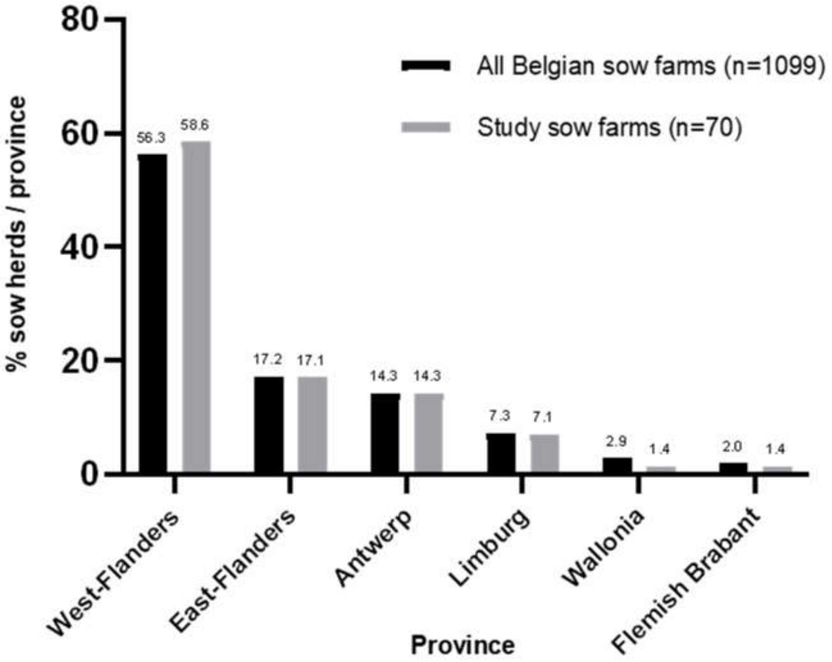

Supplementary Figure S1. Geographic distribution of the study sow farms (n=70) compared to the geographic distribution of Belgian sow farms (n=1099).

Percentage of study farms and Belgian sow farms are shown for each province. Minor differences (+2.5%, -0.1%, 0%, -0.2%, -1.5% and -0.6%) in percentage of sow herds (capacity >150 sows) per province in Belgium were observed between the study sow farms (n=70) and the total of Belgian sow farms (n=1099).

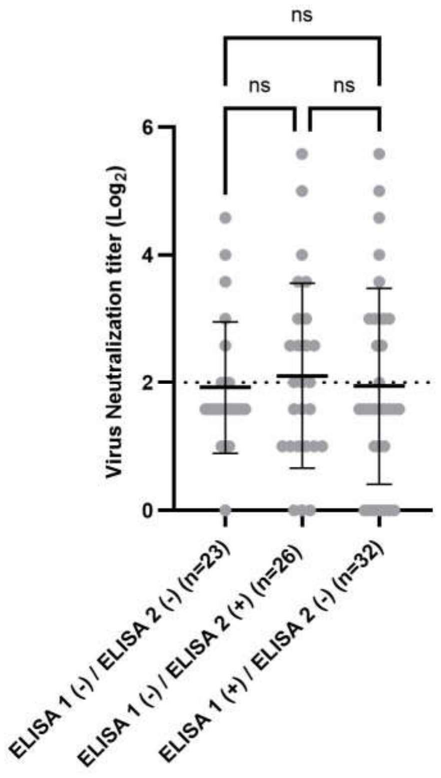

Supplementary Figure S2. Quantification of neutralizing antibodies (NAbs) against the Porcine Reproductive and Respiratory Syndrome virus (PRRSv)-1 DV strain by means of a Virus Neutralization assay (VN) in a selection of 81 ELISA 1 (IDEXX PRRS X3) and/or ELISA 2 (CIVTEST SUIS PRRS E/S) seronegative sows, originating from 37 Belgian sow herds that practice routine PRRSv vaccination.

Individual VN titers (Log2) are shown for a selection of 23 ELISA 1 and ELISA 2 seronegative sows, 26 ELISA 1 seronegative / ELISA 2 seropositive sows and 32 ELISA 1 seropositive / ELISA 2 seronegative sows. Error bars indicate the mean VN titer (Log2) and standard deviation. No significant differences in mean VN titer were observed between the different groups.
