# Supplementary figures and images for: Porcine Reproductive and Respiratory Syndrome virus (PRRSv): A Cross-Sectional Study on ELISA Seronegative, Multivaccinated Sows
Source: Viruses. 2022 Aug 31;14(9):1944. doi: 10.3390/v14091944 (PMC9501492; doi:10.3390/v14091944)

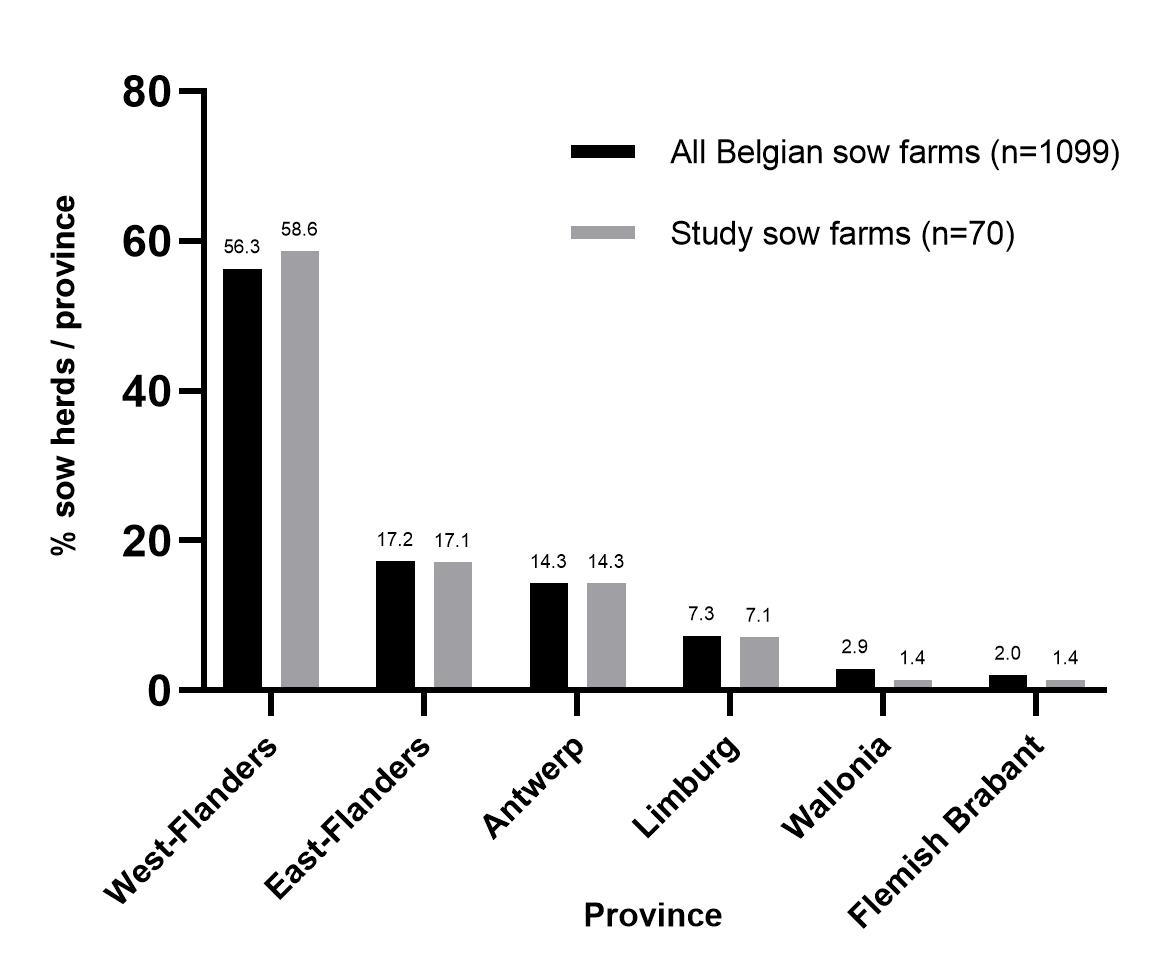

Supplement: Supplementary file 1 [file viruses-14-01944-s001.zip › Supplementary Figure S1.jpg]

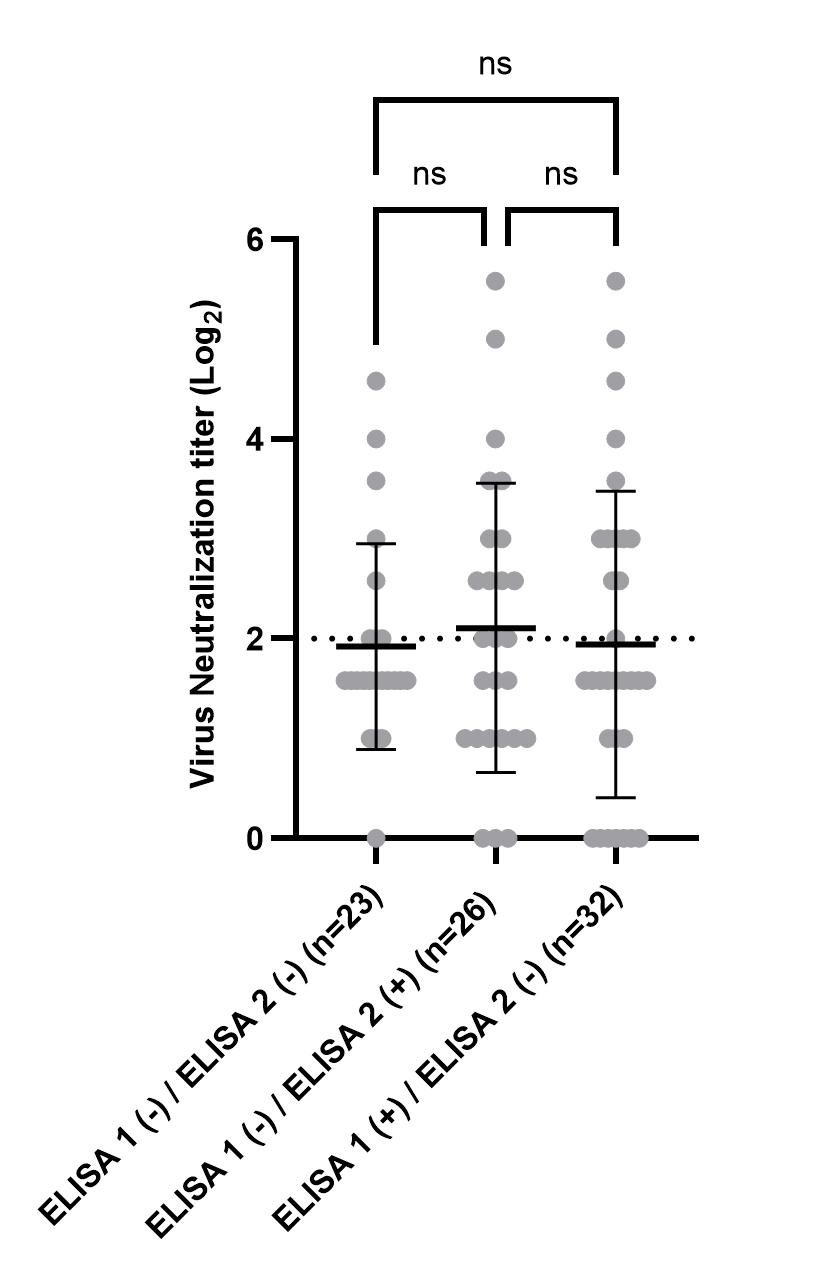

Supplement: Supplementary file 1 [file viruses-14-01944-s001.zip › Supplementary Figure S2.jpg]
